# Long Island Veterinary Society

**Published:** 1890-04

**Authors:** D. S. Breslin

**Affiliations:** Secretary; 94 Adam Street, Brooklyn, N. Y.


*Long Island Veterinary Society.*—A regular meeting of the Long Island Veterinary Society was held on March 19th, at No. 74 Adam Street, the President, Dr. George H. Berns, in the chair.

The following members were present: Drs. Berns, R. R. Bell, McLean, Pendry, Bowers, Breslin, Buckley, Jamieson, Newman, Atchison.

The minutes of the previous meeting were read and approved.

For the Board of Censors, the chairman, Dr. R. R. Bell, stated that the only business was the proposition for membership in the Society of Dr. James McKee, who was proposed by Dr. R. A. McLean. He reported as follows:

*To the President of the Long Island Veterinary Society:*

The Board of Censors desire to report that the only business before them this month is an application for membership of Dr. James McKee. We have investigated this application, and are satisfied that the applicant is a graduate of veterinary medicine; we have, however, to report adversely upon his application upon the ground that he is not a resident of Long

Island, nor a Long Island practitioner; and submit the following correspondence, upon which this report is based.

Respectfully submitted,

(Signed) ROSCOE R. BELL, *Chairman*,
THOMAS M. BUCKLEY,
W. H. PENDRY.

The correspondence requested from Dr. McKee his proper address, which he gave as Stapleton, S. I.

Dr. R. A. McLean thought the report was an insult to him, as he had proposed Dr. McKee for membership in the Society, giving his address as Nos. 14 and 16 Nevins Street, Brooklyn. The committee should have taken his word, but from their report it would look to him as if personal reasons entered the matter to keep Dr. McKee from membership in the Society.

Dr. R. R. Bell, chairman of the Board, said he had no personal animosity against the gentleman proposed, nor had any other member of the Board, but as chairman of the Board he simply carried out the duty he was elected to perform. His duty was to collect the facts in the case to the best of his ability, and from his endeavors his report is based.

A motion was made and seconded to adopt the report.

Dr. R. A. McLean called for the ayes and nays.

The ayes and nays being taken before the vote was announced by the chairman, Dr. R. A. McLean requested permission to withdraw the proposition of Dr. McKee, which was granted, after which Dr. McLean tendered his resignation as a member of the Society, and withdrew.

It was moved and seconded that the resignation of Dr. McLean be accepted. Carried.

The Committee on Army Legislation, Dr. W. H. Pendry, chairman, reported that they had communicated with Dr. Huidekoper, chairman of the Military Committee of the U. S. Veterinary Medical Association, stating that the veterinarians now in the U. S. service had a vested right in their positions, and should be protected in them by all bills introduced, and if they were not, opposition might be expected.

Dr. Huidekoper had answered, stating that the Military Committees of Congress seemed very favorably disposed to do something for a veterinary service, but still he did not believe they would recommend the appointments of over fifteen officers, nor that they would create an independent department. Also that the Military Committees had been shown copies of all proposed bills, and would undoubtedly recommend all that they think can pass. That his idea was to secure all that can be got, however little, as a nucleus.

The committee believed that the acceptance of the whole of Section VI, of the Long Island Bill, would overcome all opposition, and that it was still time to have it reintroduced.

A motion was made and carried that the society continue its confidence in the chairman of the committee, and give him full power to act for the society in the matter.

The next order of business being reading of papers, Dr. George F. Bowers, the essayist for the evening, read an interesting paper entitled

"Something Practical."[1] This paper was followed by quite a discussion, after which a vote of thanks was given to the essayist for his paper.

Dr. Bowers, as the representative of the society on the board of examiners to examine applicants for the practical prize of the American Veterinary College, reported that he met his colleagues of the board and that they decided to examine the applicants upon cases furnished in the 7th Avenue car stables; he reported that an intelligent body of men presented themselves for examination, and after a thorough and impartial examination they selected the prize man and forwarded his name to the dean of the faculty.

Communications from Prof. Huidekoper and Prof. Liautard were read, the latter containing an offer of a reprint of the proceedings of the society from the *American Veterinary Review*, at a nominal cost, providing the full proceedings of the society, including papers read, were furnished to the editor.

The secretary was instructed to communicate with Prof. Liautard requesting him to give the approximate cost of about fifty reprints of the proceedings from the *Review*.

The secretary was also instructed to communicate with Dr. R. A. McLean, requesting him to return to the society the manuscript of Dr. R. R. Bell's paper on "Azoturia," which had been read before the society. The chair appointed as essayist for the April meeting Dr. R. R. Bell. The meeting then adjourned.

D. S. BRESLIN, *Secretary*.

94 ADAM STREET, BROOKLYN, N. Y.

